# Dodine an effective alternative to copper for controlling *Venturia oleaginea*, the causal agent of pea-cock eye disease, in highly infected olive trees

**DOI:** 10.3389/fpls.2024.1369048

**Published:** 2024-03-07

**Authors:** Leen Almadi, Tommaso Frioni, Daniela Farinelli, Andrea Paoletti, Nicola Cinosi, Adolfo Rosati, Chiaraluce Moretti, Roberto Buonaurio, Franco Famiani

**Affiliations:** ^1^ Dipartimento di Scienze Agrarie, Alimentari e Ambientali, Università degli Studi di Perugia, Perugia, Italy; ^2^ Dipartimento di Scienze delle produzioni vegetali sostenibili, Università Cattolica del Sacro Cuore, Piacenza, Italy; ^3^ Consiglio per la Ricerca in Agricoltura e l’analisi dell’Economia Agraria, Centro di Ricerca OLIVICOLTURA, Frutticoltura e Agrumicoltura (CREA–OFA), Spoleto, Italy

**Keywords:** dodine, copper oxychloride, *Olea europaea* L., peacock eye disease, *Venturia oleaginea*

## Abstract

A trial was carried out in central Italy in an olive orchard of cultivar Moraiolo, highly infected by *Venturia oleaginea*. The aim of the investigation was to evaluate the effects of autumn and spring applications of copper oxychloride or dodine to control the disease. Non treated trees were used as the control. The effects of the fungal attacks on leaves and inflorescence development confirmed the high susceptibility of the cultivar Moraiolo to the disease. The results show that in trees heavily infected, but with most of the infected leaves at the early stage of the disease (asymptomatic phase), treatments with dodine had a curative effect, with consequent reduction in the appearance of symptomatic leaves and defoliation with respect to the control or copper-treated trees. The use of dodine against the autumnal attacks of *V. oleaginea* allowed most of the old leaves to be maintained until the new ones had formed, which is important for the growth processes during the early part of the growing season. Overall, the results indicate that to efficiently control the pathogen using copper compounds, treatments must start soon after the beginning of the attack and be repeated in order to maintain the infection at a low level. Dodine can be efficiently used if there is a great increase in infected leaves. The use of dodine to solve particular situations and not for normal repeated use is regulated by the fact that in some countries, Italy included, protocols for integrated pest management allow only one dodine treatment/year.

## 1 Introduction

Olive leaf spot caused by *Venturia oleaginea* (Castagne) Rossman & Crous, also known as peacock eye disease, is widespread in all countries where olive (*Olea europaea* L.) is cultivated (Agosteo and Schena, 2011; Buonaurio et al., 2023). It causes defoliation with negative consequences on vegetative growth and yield (Agosteo and Schena, 2011; Buonaurio et al., 2023). Recently, negative effects of defoliation caused by *V. oleaginea* on inflorescence development and fruit set were reported by Issa et al. (2019).

The damage caused by the pathogen is highly correlated to environmental and cultural conditions. Moist areas, rainy years, and densely planted and poorly aerated orchards are all situations that increase the susceptibility of trees to *V. oleaginea* (Issa et al., 2019; Buonaurio et al., 2023). Olive varieties show different degrees of resistance (Agosteo and Schena, 2011; Buonaurio et al., 2023). Moreover, the nutritional status of the tree can also affect its susceptibility, which increases with excessive nitrogen and potassium deficiency (Trapero and Blanco, 2010; Buonaurio et al., 2023).

Copper compounds are the most commonly used fungicides to control *V. oleaginea*. They prevent its attack on healthy leaves and cause infected ones to fall. Copper enters into the infected leaves through the openings in the cuticle caused by the pathogen during its evasion, provoking phytotoxicity and consequent leaf drop. This is beneficial because it reduces the inoculum and consequently new infections (Graniti, 1993; Trapero and Blanco, 2010). Dodine is also used to control the fungus and, due to its cytotropic penetration, it has a curative action on diseased leaves (Iannotta et al., 2002; Obanor et al., 2008). However, in a greenhouse trial carried out in New Zealand to evaluate its effectiveness, dodine was not more effective than copper sulphate (Obanor et al., 2008) and similar results were also obtained in Italy (Iannotta et al., 2002; Belfiore et al., 2014).

Since copper is a heavy metal, there is increasing concern about its use. In fact, the European Union has reduced the use of metallic copper to a maximum of 28 kg ha^-1^ over a 7-year period (on average 4 kg ha^-1^ year^-1^) and further restrictions are expected in the near future. Applications of chemicals containing a low amount of copper is a possible solution. Adawi et al. (2022) documented good protection against olive spot disease using copper complexed with lignosulphonic and gluconic acids (Disper Cu Max^®^) and a self-defense inducer (Disper Broton GS^®^), which have a metallic Cu content five and ten times lower than copper hydroxide, respectively. However, copper-free control strategies should be optimized. In this regard, further knowledge on the effectiveness of dodine would be very helpful to better understand the potential of its use in controlling *V. oleaginea* and to define its best use. Therefore, the aim of this study was to evaluate the effects of dodine applications in highly infected olive trees, using non-treated trees and copper-treated trees for comparison.

## 2 Materials and methods

### 2.1 Environment, orchard characteristics and cultural practices

The trial was carried out in, 2014-2015, in central Italy, near Spello (PG). The area is characterized by an annual average temperature of 13.6 ± 6°C and rainfalls of 700-1000 mm/year, mainly distributed from Autumn to the first part of Spring (rainfalls can be considered relatively high if compared with environments where olive is cultivated in other parts of Italy and other countries). The olive orchard used for the experiment was rainfed and located on the lower part of a hill. It consisted of adult trees of the cultivar Moraiolo, trained to the vase system and spaced 5 × 5 m. The soil was managed with permanent natural green cover. Fertilization was carried out annually with nitrogen, phosphorus and potassium fertilizers. Pruning was executed annually. Monitoring and control of olive fly was carried out as necessary.

### 2.2 Treatments

The following treatments were applied: a) non-treated trees - control; b) trees treated with copper oxychloride; c) trees treated with dodine. The treatments were applied with an atomizer in December, 2014 and April, 2015.

The copper oxychloride contained 32% metallic copper and was used at the dosage of 350 g hl^-1^ of water. The dodine fungicide contained 355 g l^-1^ of active ingredient and was used at the dosage of 150 ml hl^-1^ of water. Dodine, 1-dodecylguanidinium acetate, is an acetate salt derived from the reaction of 1-dodecylguanidine and acetic acid. It is used as a fungicide for the control of casual agents of scab, leaf spot and other foliar diseases in several species, including, apple, cherry, olive, pear, peach, strawberry and walnut (Lewis et al., 2016). Both treatments (dodine and copper) were applied with a volume of solution of, 1000 l/ha; the dosage used is that recommended by the producers and normally used with these compounds.

For each treatment, six representative trees were used as replicates.

### 2.3 Measurement of defoliation

On each tree, 8 branches were selected and labeled. At the beginning of the trial (December, 2014) leaves grown in, 2013 had all fallen off, whereas those grown in, 2014 were on the branches and easy to recognize because they had buds corresponding to their insertion on the shoot. The number of leaves grown in 2014 and the number of nodes were recorded at the beginning of the trial. The leaves were then periodically (about every 20 days) counted to determine the number that had fallen (defoliation) during the season. Defoliation is expressed as the percentage with respect to the total number of leaves counted in December, 2014.

### 2.4 Level of *V. oleaginea* infection on asymptomatic leaves

On the same shoots, the number of symptomatic leaves (attacked by *V. oleaginea* and showing classical lesions) were also counted. The level of infection on asymptomatic leaves grown in 2014 was also determined at the same dates as the labeled branches. This was done using samples of leaves (about 50 leaves/tree) collected in the parts of the canopy close to the labeled branches. The leaves were immersed in one liter of a 5% NaOH solution, obtained by dissolving 50 g of soda in 1 liter of distilled water, which was warmed to 65-70°C. After 2.5-3 min the leaves were removed from the NaOH solution and the number of spots that appeared, indicating lesions caused by the fungus, was recorded.

Observations were also carried out on the new leaves developed on the labelled shoots during the 2015 season. In these, percentages of symptomatic and asymptomatic infected leaves were calculated with respect to the total number of leaves produced up to the date of the field observation. Just before flowering (white stage of development), 30 inflorescences/tree were collected and weighed.

### 2.5 Statistical analysis

Data are presented as means ± standard error or were statistically analyzed by ANOVA according to a completely randomized design and the averages were compared by using the Student–Newman–Keuls Test.

## 3 Results

**Figure 1:**
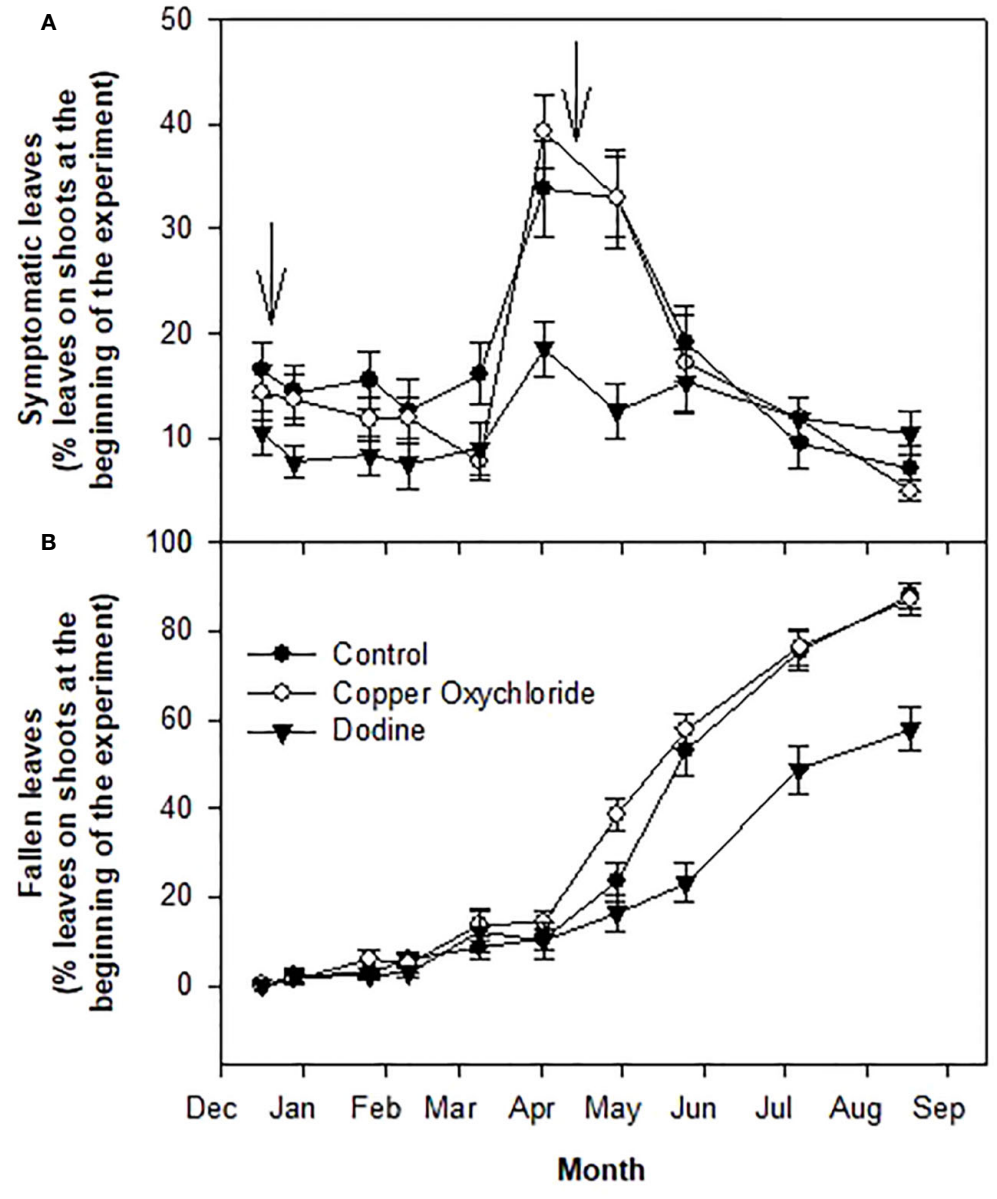
Effects of treatments on percentages of symptomatic infections of *Venturia oleaginea* **(A)** and defoliation **(B)** of leaves grown in 2014 and still present in 2015. Arrows indicate treatments with fungicides. Bars represent the standard error.

**Figure 2:**
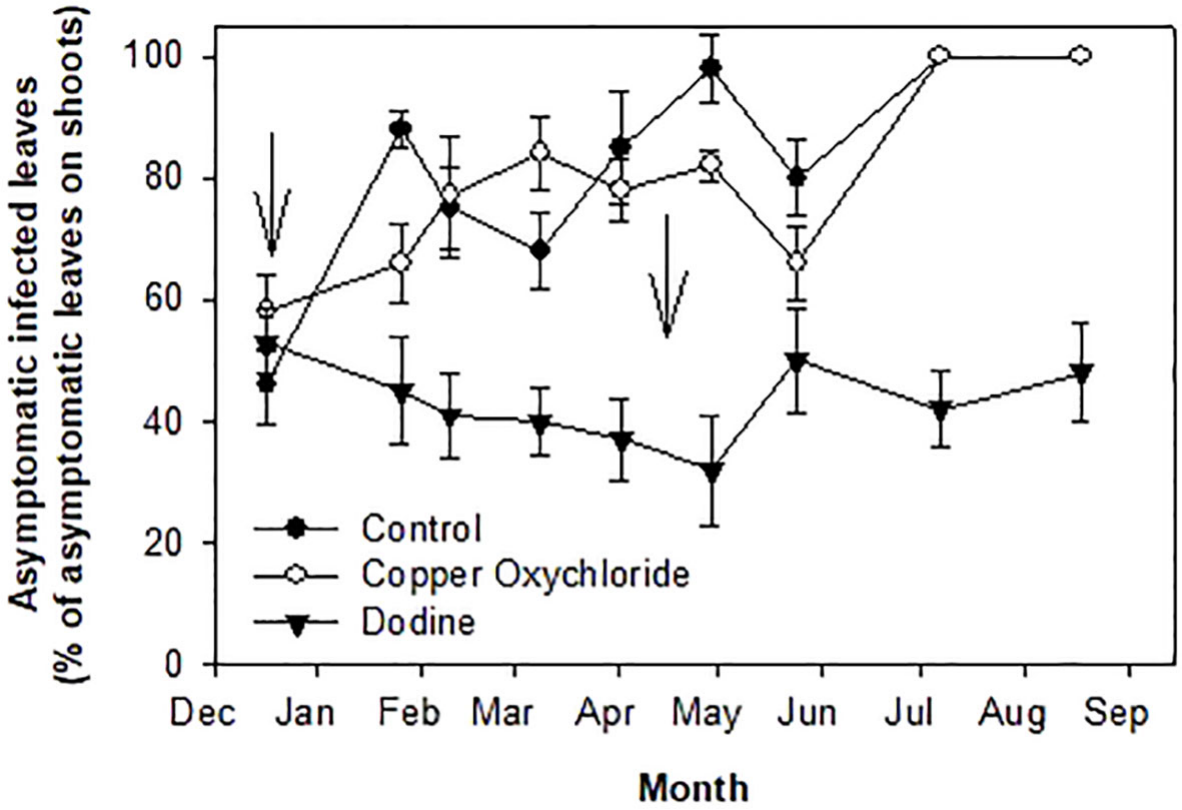
Effects of treatments on percentages of *Venturia oleaginea* infected but asymptomatic leaves grown in 2014 and still present in, 2015. Arrows indicate treatments with fungicides. Bars represent the standard error.

At the beginning of the trial (mid-December, 2014), about 15% of the leaves were symptomatic (**Figure 1A**) and about 50% of the asymptomatic ones were infected according to the NaOH test (**Figure 2**), giving a total of 65% of infected leaves.

**Figure 3:**
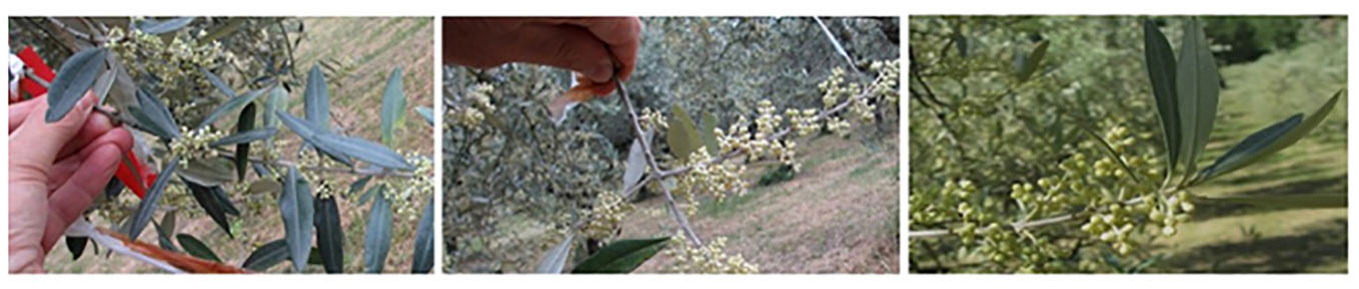
Comparison of shoots of dodine treated trees (left) with the most damaged shoots of control (center) and copper treated trees (right) at the end of May.

As reported in **Figure 1A**, the percentage of symptomatic leaves remained constant up to the beginning of March, 2015. Then, in the period April-May, 2015, it greatly increased (up to 35-40%) only in the control and copper-treated trees, but not in the dodine-treated trees, where slight increases were recorded (up to about 20%) (**Figure 3**). In parallel, starting at the beginning of April, 2015, the percentage of fallen leaves increased intensely in control trees and, especially, in copper-treated trees, whereas a lesser increase was observed in dodine-treated trees (**Figure 1B**). At the end of the experiment, defoliation was around 90% in the control and copper-treated trees and 55% in the dodine-treated trees (**Figure 1B**).

The percentage of asymptomatic infected leaves on the dodine-treated trees had no substantial variations, with a tendency to decrease up to the end of March and then to increase to constant values of 40-50% (**Figure 2**). In contrast, control, and copper-treated trees showed increasing values, reaching 100% in July.

**Figure 4:**
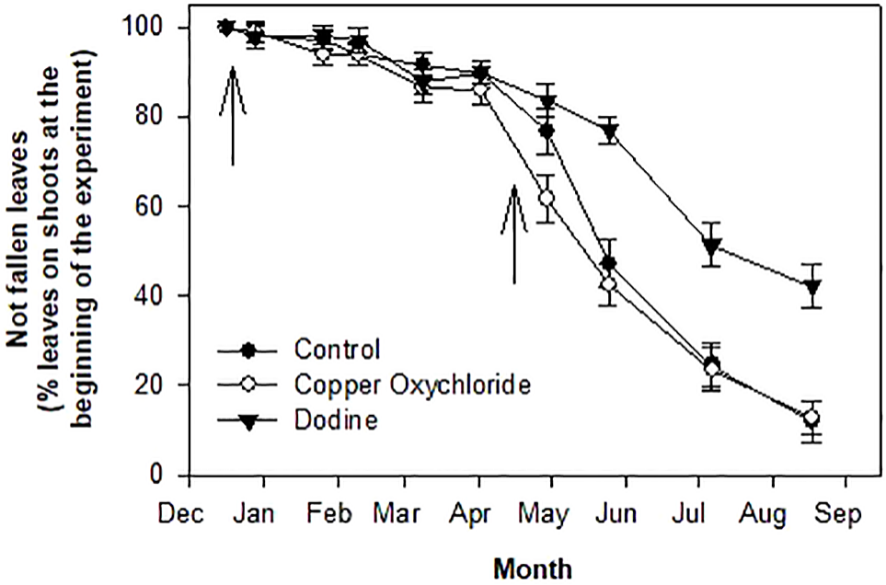
Effects of treatments on percentages of not fallen leaves. Arrows indicate treatments with fungicides. Bars represent the standard error.

Dodine treatments ensured that most of the leaves remained on the trees during the entire spring season: about 85% at the end of April, 75% at the end of May and 65% at the end of spring (third decade of June), whereas in the same periods the percentages were about 75, 45 and 30 in the control and 60, 40 and 30 in the copper-treated trees (**Figure 4**). At the end of the experiment (mid-August) the values were about 45% in the dodine-treated trees and 10% in both the control and copper-treated trees (**Figure 4**).

New leaves grown in 2015 were not infected in the dodine- and copper-treated trees, whereas leaves of the control trees were infected (**Table 1**). It is noteworthy that, dodine-treated trees had the heaviest inflorescences.

**Table 1:** Effects of treatments on inflorescence growth/weight and percentages of new leaves grown in, 2015 which showed peacock eye disease at the end of the experiment (August, 2015).

Treatment	Inflorescence weight (mg/inflorescence)	Symptomatic leaves (%)	Asymptomatic infected leaves (%)	Symptomatic + asymptomatic infected leaves (%)
**Control**	206 b	38.0 a	10.2 a	48.2 a
**Copper oxychloride**	190 b	0.0 b	0.0 b	0.0 b
**Dodine**	230 a	0.0 b	0.0 b	0.0 b

In each column, means followed by the same letter are not significantly different at *p* = 0.05.

## 4 Discussion

Severe and recurrent attacks of *Venturia oleaginea* in olive trees provoke intense defoliation and poor growth and dieback of defoliated branches with marked negative impact on tree productivity (Buonaurio et al., 2023). In case of high levels of infection, application of copper compounds, which is the widespread control strategy, is not fully effective in the short-medium period, because it causes diseased leaves to drop. Therefore, in olive leaf spot control, it is essential to find alternative strategies able to significantly reduce the fungal inoculum in the orchard and, at the same time, avoid/reduce leaf loss. Hence, the curative cytotropic-translaminar fungicide dodine was tested as an alternative to copper compounds. This was applied in an olive orchard with a high level of infection selected according to olive variety and cultivation site.

The high levels of defoliation recorded in early spring confirmed that Moraiolo is a very susceptible cultivar to *V. oleaginea* (Raggi and D'Armini, 1996). The importance of resistance/susceptibility of olive varieties to the pathogen in determining the level of attack is well known (Agosteo and Schena, 2011; Sanei and Razavi, 2011; Rongai et al., 2012; Abuamsha et al., 2013; Buonaurio et al., 2023). The high levels of infections observed at the beginning of the experiments in symptomatic and asymptomatic leaves (15% + 50% = 65% of infected leaves) is due to the high susceptibility of the Moraiolo cultivar, the abundant rainfalls recorded in 2014 and 2015 and the orchard position which is located at the base of a hill where high relative air humidity occurs. These are all factors known to promote fungal attack (Obanor et al., 2008; Trapero and Blanco, 2010; Salman et al., 2011; Sanei and Razavi, 2011). The observed percentage of diseased leaves (65%) at the beginning of the trial is very high, considering that a value of 20% can be used as a threshold to execute treatments to keep the disease under control and keep the trees in a suitable physiological state (Almadi et al., 2024).

There were significant differences in the effects of the treatments. Dodine was much more effective than copper in limiting the damage caused by the fungal attack. This can be explained by the preventive, curative and eradicating action of dodine, which, actually, was able to effectively counteract *V. oleaginea* infections already underway and protect the leaves from new attacks. It is noteworthy that up to now no resistant *V. oleaginea* strains have been reported. The first treatment with dodine in mid-December was able to stop new infections of *V. oleaginea* up to the time of the second treatment in April (**Figure 2**). Copper oxychloride limited new infections up to February, after which there was an increase in infections (**Figure 1A**). It is likely, that the rain during the winter season washed the copper off the leaf surface, eliminating its protective effect, whereas dodine which penetrates the leaves ensured longer protection. It is interesting to analyze the results with respect to the new vegetation. In the site investigated, the growth of new shoots starts in mid-April and lasts up to mid-July (Farinelli et al., 2011). Successively, there could be a slight new growth in September, but it is usually very poor. Therefore, the formation of new leaves mainly occurs between mid-April and mid-July, with the highest intensity in May-June. This means that in trees treated with dodine, even in years with heavy attacks of *V. oleaginea* in autumn, which cause high defoliation in the successive spring, it is possible for most of the old leaves to be maintained until the new ones have been formed, whereas this was not the case in the control and copper-treated trees. At the end of the experiment, in dodine-treated trees there were still about 45% of the leaves versus about 10% in control and copper-treated ones. This is a very good result because, even though the life span of an olive leaf is up to 3 years, most (that is more than 50%) of the leaves naturally drop in their second year during the new growth and when the leaves become shaded (Lavee, 1996). Therefore, considering this natural defoliation, the control exerted by dodine on the disease symptoms was very high.

In other field studies, the use of dodine and copper oxychloride showed similar effects (Iannotta et al., 2002; Belfiore et al., 2014; Adawi et al., 2022). The differences in the results obtained in the present study with respect to those of Iannotta et al. (2002) and Belfiore et al. (2014) may be due to the heavy but still mainly asymptomatic attack of the trees in our study. Indeed, at the beginning of the experiment the attack was at an initial stage of disease development (only 15% of the leaves were symptomatic). In this situation, dodine was able to cure the attacked leaves, keeping them from dropping or slowing down their drop. Instead, copper oxychloride, which has a preventive action, protected the healthy leaves from infections, but caused the attacked ones to drop. This last effect was evident after the second treatment in April and explains the different pattern of defoliation observed in dodine-treated trees with respect to the copper-treated ones. Comparing the effects of the first and the second treatment, it is possible to see that defoliation caused by copper occurs when treatments are performed on leaves with advanced levels of infection. The acceleration of leaf drop caused by copper treatments is known (Agosteo and Schena, 2011) and our results confirm this.

*V. oleaginea* exerts its negative effect by infecting the leaves, causing defoliation. This can explain the differences in the development of inflorescences among the different treatments. In a study on the physiology of infected leaves, Proietti et al. (2002) showed that the disease reduces photosynthetic leaf activity. Therefore, the higher number of infected symptomatic leaves and defoliation in the control and copper-treated trees may have caused a great reduction in the production of assimilates, which negatively affected the formation of inflorescences and thus their growth. Indeed, the inflorescences were heavier on dodine-treated trees, which, with respect to the control and copper-treated trees, greatly reduced the level of infection and defoliation. These results are in agreement with a previous study showing a negative effect of infections and defoliation caused by *V. oleaginea* on inflorescence growth (Issa et al., 2019). Both dodine and copper oxychloride were effective on young leaves because they both acted by preventing *V. oleaginea* attacks.

In conclusion, the results show that in trees heavily attacked by *V. oleaginea*, but with most of the infected leaves being asymptomatic, treatments with dodine reduced new infections, the appearance of symptomatic leaves and defoliation with respect to the control or copper-treated trees. The use of dodine against the autumnal attack of *V. oleaginea*, which causes heavy defoliation in the following spring, allowed most of the old leaves to be maintained until the new ones had formed. This is important as it supports the growth processes which occur in the early part of the growing season, as demonstrated by the greater growth of inflorescences in dodine-treated trees with respect to the control and copper-treated ones. Overall, the results indicate that to efficiently control *V. oleaginea* with copper compounds, as they have no curative effect and accelerate the dropping of infected leaves, treatments must be started soon after the beginning of the attack, revealed by using the NaOH test (Buonaurio et al., 2023) and repeated in order to keep the infection at a low level. Dodine can be efficiently used if there is a great increase in infected leaves. Dodine should be used to solve particular problems and not for normal use as in some countries, such as Italy, protocols for integrated pest management allow only one dodine treatment/year. However, in the case of very heavy autumn infections, treatments with dodine can be done both in autumn and in early spring the following year, with a synergistic effect.

In conclusion, when there is a high level of peacock eye infection, dodine is a very effective fungicide to control it. Due to its curative mode of action, it can ensure a greater flexibility in the control of this important disease, allowing a significant reduction of the inoculum, without causing immediate leaf loss.

## Data availability statement

The raw data supporting the conclusions of this article will be made available by the authors, without undue reservation.

## Author contributions

LA: Data curation, Formal Analysis, Investigation, Methodology, Validation, Writing – original draft. TF: Data curation, Formal Analysis, Investigation, Methodology, Validation, Writing – original draft. DF: Investigation, Methodology, Validation, Writing – original draft. AP: Data curation, Investigation, Methodology, Validation, Writing – original draft. NC: Writing – original draft. AR: Investigation, Methodology, Validation, Writing – original draft. CM: Investigation, Methodology, Supervision, Validation, Writing – review & editing, Writing – original draft. RB: Conceptualization, Methodology, Supervision, Validation, Writing – review & editing, Writing – original draft. FF: Conceptualization, Funding acquisition, Methodology, Project administration, Supervision, Validation, Writing – original draft, Writing – review & editing.
